# Disorders of Body Weight, Sleep and Circadian Rhythm as Manifestations of Hypothalamic Dysfunction in Alzheimer’s Disease

**DOI:** 10.3389/fncel.2018.00471

**Published:** 2018-12-05

**Authors:** Abigail J. Hiller, Makoto Ishii

**Affiliations:** ^1^Feil Family Brain and Mind Research Institute, Weill Cornell Medicine, Cornell University, New York, NY, United States; ^2^Department of Neurology, Weill Cornell Medicine, Cornell University, New York, NY, United States

**Keywords:** hypothalamus, obesity, diabetes, sleep, circadian rhythm, dementia, amyloid-beta, tau

## Abstract

While cognitive decline and memory loss are the major clinical manifestations of Alzheimer’s disease (AD), they are now recognized as late features of the disease. Recent failures in clinical drug trials highlight the importance of evaluating and treating patients with AD as early as possible and the difficulties in developing effective therapies once the disease progresses. Since the pathological hallmarks of AD including the abnormal aggregation of amyloid-beta (Aβ) and tau can occur decades before any significant cognitive decline in the preclinical stage of AD, it is important to identify the earliest clinical manifestations of AD and elucidate their underlying cellular and molecular mechanisms. Importantly, metabolic and non-cognitive manifestations of AD such as weight loss and alterations of peripheral metabolic signals can occur before the onset of cognitive symptoms and worsen with disease progression. Accumulating evidence suggests that the major culprit behind these early metabolic and non-cognitive manifestations of AD is AD pathology causing dysfunction of the hypothalamus, a brain region critical for integrating peripheral signals with essential homeostatic physiological functions. Here, we aim to highlight recent developments that address the role of AD pathology in the development of hypothalamic dysfunction associated with metabolic and non-cognitive manifestations seen in AD. Understanding the mechanisms underlying hypothalamic dysfunction in AD could give key new insights into the development of novel biomarkers and therapeutic targets.

## Introduction

Alzheimer’s disease (AD) is the leading cause of dementia in the elderly and remains an incurable and devastating neurodegenerative disease (Alzheimer’s Association, 2018). Though the exact pathogenesis of AD remains unclear, abnormal accumulation of amyloid-beta (Aβ) peptides and the microtubule-associated protein tau over time leads to neuronal and synaptic dysfunction and the neuropathological hallmarks of extracellular Aβ plaques and neurofibrillary tau tangles (Sala Frigerio and De Strooper, 2016). While cognitive symptoms are the most prominent feature of AD, they are now recognized as a late manifestation. Aβ and tau pathology can be detected by cerebrospinal fluid (CSF) analysis or positron emission tomography (PET) imaging in the preclinical stage of AD, decades prior to the cognitive impairment seen in mild cognitive impairment (MCI) or dementia (Dubois et al., 2016). Notably, various metabolic and non-cognitive manifestations of AD including weight loss and sleep and circadian rhythm disorders can precede the cognitive decline (Ishii and Iadecola, 2015). The hypothalamus, which serves as the brain’s integrator of peripheral metabolic signals and houses the central circadian pacemaker, the suprachiasmatic nucleus (SCN), is uniquely positioned to mediate many of these non-cognitive changes. Importantly, both Aβ and tau pathology have been found in the hypothalamus of AD brains (Table 1). Moreover, non-cognitive symptoms can worsen with disease progression and are associated with increased morbidity and mortality in AD, highlighting the importance of hypothalamic dysfunction in AD. In this review article, we discuss recent developments in understanding the relationship between AD pathobiology and select metabolic and non-cognitive manifestations that are putatively linked to hypothalamic dysfunction.

**Table 1:** Hypothalamus and Alzheimer’s disease (AD): select pathological and neuroimaging studies.

Study type	Hypothalamic findings
Histopathology (amyloid)	Amyloid deposition was observed in all hypothalamic nuclei by Braak Stage C (Braak and Braak, 1991) Specifically, amyloid plaques have been reported in many hypothalamic regions including the mammillary bodies, SCN, TMN, LTN, VMN and fornix (Stief, 1927; Rudelli et al., 1984; McDuff and Sumi, 1985; Stopa et al., 1999)
Histopathology (tau)	Isolated neurofibrillary tangles (NFT) was observed first in TMN at Braak Stage III and by Stage VI are widespread in TMN and LTN (Braak and Braak, 1991) NFT and tau staining have been reported in the mammillary bodies, SCN, DMN, VMN, arcuate (infundibular) nucleus and fornix (Stief, 1927; Rudelli et al., 1984; McDuff and Sumi, 1985; Saper and German, 1987; van de Nes et al., 1998; Schultz et al., 1999; Stopa et al., 1999)
MRI (volume)	Decrease in hypothalamic volume was observed by moderate AD (Callen et al., 2001) Hypothalamic atrophy was more pronounced in males than in females (Callen et al., 2004) and positively correlated with BMD loss in mild AD (Loskutova et al., 2010)
^18^FDG-PET (glucose metabolism)	Reduced glucose metabolism in the hypothalamus was observed in MCI and AD patients (Nestor et al., 2003; Cross et al., 2013)

*Abbreviations: BMD, bone mineral density; DMN, dorsomedial nucleus; FDG, fluorodeoxyglucose; MCI, mild cognitive impairment; MRI, magnetic resonance imaging; NFT, neurofibrillary tangles; LTN, lateral tuberal nucleus; PVN, paraventricular nucleus; SCN, suprachiasmatic nucleus; TMN, tuberomammillary nucleus; VMN, ventromedial nucleus*.

## Body Weight and Systemic Metabolism

### Late-Life Weight Loss: An Early Manifestation of AD

Weight loss has long been recognized as a clinical manifestation of AD and was considered a criteria consistent with the diagnosis of probable AD in the 1984 NINCDS-ADRDA work group report (McKhann et al., 1984). Importantly, weight loss in AD patients correlated with increased morbidity and mortality (White et al., 1998; Jang et al., 2015) and cortical Aβ load (Blautzik et al., 2018). Furthermore, MCI subjects who are underweight or lose weight have an increased risk for progressing to AD (Sobów et al., 2014; Joo et al., 2018). These studies collectively suggest that weight loss is an intrinsic feature of AD pathobiology.

While weight loss once dementia manifests could be attributed to impairments in appetite and eating behavior, epidemiological studies have found consistently that late-life weight loss can precede the cognitive decline in AD (Barrett-Connor et al., 1996; Buchman et al., 2005; Stewart et al., 2005; Johnson et al., 2006; Gao et al., 2011; Emmerzaal et al., 2015; Jimenez et al., 2017). Additionally, in a large community cohort study, late-life weight loss increased the risk for developing MCI regardless of mid-life body weight, suggesting that late-life weight loss is a clinical manifestation of early stages of AD regardless of mid-life metabolic risk factors (Alhurani et al., 2016). Furthermore, a recent study from the Dominantly Inherited Alzheimer Network (DIAN) found that asymptomatic carriers of gene mutations for autosomal dominant AD had significantly lower body mass index (BMI) compared to nonmutation carriers with weight loss starting more than a decade before onset of cognitive symptoms (Müller et al., 2017). Importantly, lower BMI was found to be associated with higher brain Aβ burden and lower scores on a delayed memory recall test in the asymptomatic AD mutation carriers.

Mounting evidence suggests that the adipocyte-derived hormone (adipokine) leptin is affected in AD. Leptin is produced in proportion to adiposity and serves as a critical negative afferent signal to the brain and in particular the hypothalamus to regulate body weight and systemic metabolism (Friedman, 2014). Low circulating leptin levels have been consistently found in AD subjects (Lieb et al., 2009; Bigalke et al., 2011; Khemka et al., 2014; Ma et al., 2016; Yu et al., 2018). While the underlying mechanisms for the early weight loss and low circulating leptin levels remain to be fully elucidated, dysfunction of the hypothalamus is likely to be a major driver. Compared to wild-type littermates, young transgenic mice with Aβ pathology (Tg2576 mice) prior to plaque formation or significant cognitive impairment exhibited low body weight/adiposity and low plasma leptin levels, which was associated with Aβ-mediated dysfunction of select hypothalamic neurons important for the regulation of body weight (Ishii et al., 2014). Taken together, these findings from animal and human studies raise the intriguing possibility that Aβ could interfere with hypothalamic sensors of peripheral metabolic signals such as leptin, preventing the brain from responding to signals of low body weight/adiposity resulting in pathologically low circulating leptin levels and unintentional weight loss.

Additionally, leptin is important for maintaining hippocampal structure and function (McGregor and Harvey, 2017) and exerting neuroprotective effects under a variety of neurotoxic conditions including Aβ (McGuire and Ishii, 2016). In humans, low circulating leptin levels have been associated with cognitive decline in the elderly (Holden et al., 2009) and decreased hippocampal gray matter volume (Narita et al., 2009). Similarly, studies using various transgenic mouse models of Aβ pathology have consistently found that leptin levels correlate positively with cognitive function and negatively with Aβ burden (Greco et al., 2010; Takeda et al., 2010; Pérez-González et al., 2014). Therefore, alterations in leptin signaling associated with unintentional weight loss may serve not only as a marker of early AD but may contribute to AD pathogenesis. While leptin has been the most extensively investigated adipokine in AD, a significant role for other adipokines and peripheral metabolic signals cannot be excluded (Kiliaan et al., 2014).

### Mid-Life Obesity and Diabetes: Risk Factors for Developing AD

In contrast to late-life weight loss, mid-life obesity and related comorbid conditions including insulin resistance and type 2 diabetes mellitus (T2DM) have been found in several epidemiological studies to be risk factors for cognitive decline and AD (Arvanitakis et al., 2004; Kivipelto et al., 2005; Biessels et al., 2014; McGuire and Ishii, 2016). In contrast, a large population study in the UK found that mid-life obesity decreased risk for dementia (Qizilbash et al., 2015); however, this study may have potential confounding factors including reverse causation bias and ill-defined range of mid-life (Gustafson, 2015; Kivimäki et al., 2015). While additional studies are clearly needed, the current evidence suggests that age is an important factor when considering body weight and adiposity changes in AD with mid-life obesity being a risk factor and late-life weight loss being an early manifestation of AD.

In light of the association between mid-life obesity and T2DM and AD, it has been noted that these metabolic disorders cause damage to the hypothalamus by similar mechanisms to those seen in AD (Clarke et al., 2018). Physiological consequences of obesity and T2DM, including chronic hyperinsulinemia and high circulating levels of free fatty acids have been shown to lead to hypothalamic insulin and leptin resistance (Thon et al., 2016), ER stress (Zhang et al., 2008; Mayer and Belsham, 2010) and pro-inflammatory intracellular cascades (Milanski et al., 2009) in hypothalamic neurons. Similarly, Aβ oligomers induced TNF-alpha mediated inflammation and ER stress in cultured hypothalamic neurons and the hypothalamus of mice and macaques (Clarke et al., 2015). Furthermore, an NMR-based metabolomics study of the transgenic amyloid precursor protein/presenilin 1 (APP/PS1) mouse model of Aβ pathology found that these mice had significant hypothalamic metabolic abnormalities prior to memory impairment (Zheng et al., 2018). These studies provide further support that hypothalamus dysfunction can occur early in the development of AD and is likely mediated by mid-life metabolic risk factors of obesity and T2DM.

There is also substantial evidence to suggest that obesity and T2DM related pathologies could directly promote early AD pathology. Studies in mouse models and humans have shown that hyperinsulinemia and insulin resistance can increase Aβ load by interfering with clearance mechanisms and increasing production of Aβ (Stanley et al., 2016; Ramos-Rodríguez et al., 2017; Benedict and Grillo, 2018). Additionally, obesity and T2DM lead to increased deposition of human islet APP (hIAPP or amylin) in not only pancreatic islets but in the brain parenchyma and cerebrovascular system, which may exacerbate AD pathology by causing neurotoxicity and decreased Aβ clearance (Jackson et al., 2013; Wijesekara et al., 2017). The cross-seeding of misfolded hIAPP and Aβ peptides has been hypothesized as a mechanism for shared disease pathogenesis between AD and T2DM (Moreno-Gonzalez et al., 2017). However, not all studies show that obesity and T2DM worsens AD pathology. For example, a mouse model of human tau pathology given a high-fat, high-sugar and high-cholesterol diet had no significant changes in hippocampal and cortical tau pathology (Gratuze et al., 2016).

## Sleep and Circadian Rhythm Disorders

### Sleep Disorders

Sleep disorders affect 25%–66% of AD patients and are a leading cause for institutionalization (Bianchetti et al., 1995; Moran et al., 2005; Guarnieri et al., 2012). Importantly, sleep quality in AD declines early in the disease and worsens with disease progression (Vitiello et al., 1990; Liguori et al., 2014). Furthermore, cognitively normal subjects with Aβ deposition by CSF measurements had worse sleep quality compared to those without Aβ deposition (Ju et al., 2013). This association between AD pathology and poor sleep quality has been recapitulated in multiple mouse models with increased Aβ deposition (Wisor et al., 2005; Roh et al., 2012; Sethi et al., 2015). Additionally, a single intracerebroventricular (ICV) infusion of Aβ oligomers disrupted sleep patterns in mice (Kincheski et al., 2017). Taken together, these findings provide evidence that AD pathology impacts sleep early in AD and may occur prior to the onset of cognitive symptoms.

Accumulating evidence suggests that hypothalamic dysfunction is responsible for the sleep dysfunction in AD. A recent study found reduced hypothalamic glucose uptake, as measured by ^18^F-flurodeoxyglucose PET, in AD subjects compared to non-demented control subjects, which was associated with sleep impairment and CSF AD biomarkers (Liguori et al., 2017). Evidence also exists for the involvement of specific hypothalamic nuclei in the sleep dysfunction in AD. The intermediate nucleus of the hypothalamus, the putative analog to the ventrolateral preoptic nucleus (VLPN) in rodents, contains neurons that are active in both rapid eye movement (REM) and non-REM (NREM) sleep (Chung et al., 2017; Saper and Fuller, 2017). A decrease of galanin-positive neurons in the intermediate nucleus was reported in postmortem AD brains (Lim et al., 2014). Because these neurons are active during sleep and inhibit wake-promoting neurons, loss of VLPN galanin neurons presents a potential mechanism for decreased NREM sleep and increased awakenings in AD (Saper and Fuller, 2017).

Another important hypothalamic nucleus in the regulation of sleep is the lateral hypothalamic area (LH), which contains neurons that synthesize the neuropeptide orexin (hypocretin). Orexin is critical for the maintenance of sleep-wake architecture by promoting arousal with orexin deficiency resulting in narcolepsy (Tsujino and Sakurai, 2013). In human studies, there are conflicting reports regarding orexin levels in AD with multiple studies reporting unchanged or decreased CSF and hypothalamic levels (Fronczek et al., 2012; Schmidt et al., 2013; Liguori et al., 2014). In contrast, more recent studies suggest that accumulating AD pathology is associated with increased CSF orexin levels and sleep disruption. In AD biomarker-defined MCI subjects, increased CSF orexin levels were associated with REM sleep disruption and sleep fragmentation (Liguori et al., 2016). In another study, higher CSF orexin levels were found in biomarker-defined AD subjects compared to MCI and control groups (Gabelle et al., 2017).

Substantial support also exists for sleep dysfunction worsening AD pathology and increasing the risk for developing dementia (Mander et al., 2016). A recent meta-analysis found that sleep disorders such as insomnia and sleep-disordered breathing increased the risk for developing AD (Shi et al., 2018). Prolonged sleep duration in older adults was also associated with increased development of dementia (Westwood et al., 2017). Therefore, abnormal sleep, regardless of the duration, is associated with increased dementia risk. Additionally, human and animal studies have found that poor sleep quality including deprivation can worsen AD pathology. In healthy human adults, a single night of lost sleep was associated with an increased Aβ load as measured by CSF and brain PET studies (Ooms et al., 2014; Shokri-Kojori et al., 2018). Furthermore, several studies have found various measures of poor sleep quality were associated with increased brain Aβ load in cognitively normal individuals (Spira et al., 2013; Branger et al., 2016) Consistent with these human studies, sleep deprivation or increased wakefulness in a *Drosophila* or transgenic mouse model of Aβ pathology increased Aβ burden (Kang et al., 2009; Roh et al., 2014; Tabuchi et al., 2015). The underlying mechanism behind the association between sleep and Aβ pathology has been hypothesized to be due to increased clearance of Aβ during sleep (Xie et al., 2013) or neuronal activity-dependent increases in Aβ secretion during wakefulness (Cirrito et al., 2005; Tabuchi et al., 2015). Despite some conflicting studies, the current evidence supports a bidirectional relationship where AD pathology can cause increased orexin levels and disruption of sleep, while disruption of sleep can lead to increased AD pathology.

### Circadian Rhythm Disorders and Sundowning

Closely related to sleep disorders, circadian rhythm abnormalities including disrupted day-night activity patterns are common in AD patients (Musiek et al., 2015). In particular, aggressive behaviors in AD are often temporally dependent, worsening in the afternoon and evening, in a pattern that is clinically termed Sundown Syndrome or “sundowning” (Khachiyants et al., 2011). Importantly, agitation such as seen with sundowning in AD patients can precede significant adverse outcomes including institutionalization, accelerated cognitive decline and increased caregiver burden (Canevelli et al., 2016). Yet, current strategies for managing aggressive symptoms rely on pharmacological interventions including anti-psychotics that may not target the underlying pathways affected and can have significant adverse effects (Ballard and Corbett, 2013). Therefore, understanding the underlying mechanisms of sundowning would be critical for improving the clinical care of AD patients.

The hypothalamus has long been recognized as a major regulator of both circadian rhythm and aggressive behaviors, suggesting a potential role in sundowning. Dysfunction in the hypothalamic SCN, the central pacemaker, is a likely mediator of circadian rhythm disorders in AD (Van Erum et al., 2018). In AD patients, the SCN shows increased aging-related atrophy and neurodegeneration with evidence for neurofibrillary tangle accumulation (Swaab et al., 1985; Stopa et al., 1999). Additionally, in postmortem AD brains, blunted fluctuations in circadian motor activity and increased SCN amyloid plaque burden are reported to be correlated with reduction of two central circadian neurotransmitters, vasopressin and neurotensin (Stopa et al., 1999; Harper et al., 2008; Hu et al., 2013), although one study reported no change in SCN vasopressin levels in AD (Wang et al., 2015). Similarly, the hypothalamus has been long implicated in the role of aggressive behaviors. In the early 20th century, electrical stimulation of specific regions of the hypothalamus including the LH and the VMH promoted aggression in cats (Hess and Akert, 1955). These areas of the hypothalamus have been classically identified as “attack areas, ” and their stimulation in a variety of animal species has been linked with distinct aggressive behaviors (Haller, 2013). A recent study identified a hypothalamic circuit involving projections from the SCN to the VMH that regulated the daily rhythm in aggression propensity of male mice (Todd et al., 2018), suggesting that disruption of this hypothalamic circuit could lead to sundowing in AD.

Molecular and genetic studies in animal models further support the hypothalamus and in particular the SCN playing a central role in circadian rhythm disorders associated with AD pathology. A mouse model of Aβ pathology was found to have dampened SCN excitability rhythms, concurrent with circadian-associated behavioral disturbances and reduced daytime A-type potassium currents (Paul et al., 2018). In contrast, several Aβ mouse and *Drosophila* models exhibit circadian behavioral abnormalities despite normal central clock function, suggesting that Aβ-related circadian abnormalities may also stem from a “central clock output failure” in which the SCN fails to entrain brain-resident and peripheral clocks (Chauhan et al., 2017).

## Conclusions

**Figure 1:**
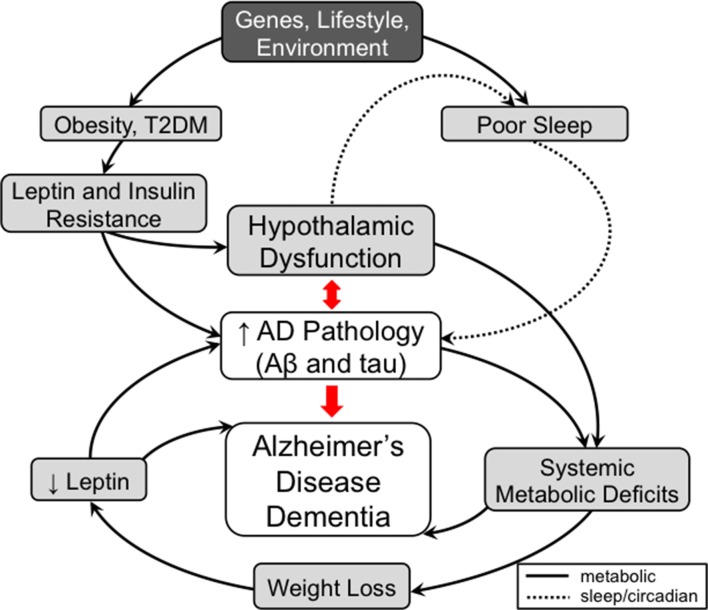
A model for the contribution of metabolic and non-cognitive factors (e.g., sleep/circadian rhythm) in the pathogenesis of Alzheimer’s disease (AD). In mid-life, obesity and type 2 diabetes mellitus (T2DM) are risk factors for AD. These conditions are associated with high circulating insulin and leptin levels leading to the development of hypothalamic dysfunction, including insulin and leptin resistance, as well as to worsening AD pathology directly. Development of hypothalamic insensitivity to peripheral metabolic signals in mid-life sets the stage for exacerbation of metabolic dysregulation in late-life AD, when accumulation of AD pathology can lead to further neuronal injury. A possible model to explain the correlation between late-life weight loss and AD posits that AD pathology-mediated neuronal injury in the hypothalamus leads to a hypermetabolic (catabolic) state, which results in weight loss and a pathologically low leptin state. As leptin has possible roles as a neuroprotective factor and a regulator of hippocampal structure and function, deficiency in leptin signaling could also contribute to cognitive impairment independent of hypothalamic signaling. Similar to metabolic dysfunction, sleep disorders play a role in AD pathogenesis. Poor sleep quality in mid-life has been associated with increased AD pathology. In late-life, hypothalamic dysfunction caused by AD pathology contributes to the sleep dysfunctions seen in AD. The worsening sleep disorders would then feed forward into the development of further AD pathology and eventually dementia. Therefore, disorders of metabolism and non-cognitive (e.g., sleep/circadian rhythm) factors mediated by hypothalamic dysfunction are both early risk factors and manifestations of AD that can contribute in a feed-forward manner that ultimately results in AD dementia. Solid lines represent metabolic pathways and dashed lines represent pathways related to sleep and circadian rhythms.

We have briefly reviewed select recent findings on the metabolic and non-cognitive manifestations of AD that can occur before the cognitive decline and focused specifically on disorders of body weight, sleep and circadian rhythm. We provide evidence that these metabolic and non-cognitive manifestations of AD are due to hypothalamic dysfunction caused by AD pathology and can be bidirectional and feed-forward in nature (Figure 1). Furthermore, while body weight and sleep/circadian rhythm may appear to act independently from each other, they often share common neurotransmitters (e.g., orexin, galanin) and brain regions (e.g., VMH, LH) in the hypothalamus, which can be modulated by peripheral circulating factors such as leptin and glucose (Fang et al., 2012; Tsujino and Sakurai, 2013; McGuire and Ishii, 2016). Therefore, seemingly disparate clinical manifestations of AD may be due to alterations of common hypothalamic pathways affected early in AD.

Despite recent advances, there are significant gaps in our knowledge. The hypothalamus is a complex brain region comprised of numerous distinct molecular cell types with each potentially a part of multiple different pathways. While several studies using a candidate-based approach have identified select individual cell types affected by AD pathology (Ishii et al., 2014; Clarke et al., 2015), the exact cell types affected in the hypothalamus are not known. Therefore, large unbiased molecular screens such as with Drop-Seq and similar approaches will likely be needed (Campbell et al., 2017). Additionally, once the cell types affected by AD pathology are identified, the exact cellular mechanisms leading to the dysfunction of those neurons and whether they are similar to those seen in more extensively studied brain regions such as the hippocampus need to be elucidated. Finally, any mechanistic studies in cellular or animal models needs to be validated and verified in carefully conducted AD biomarker-defined human studies.

While cognitive manifestations have deservedly received the bulk of the attention in AD research, non-cognitive manifestations are often correlated with disease progression, increased morbidity including institutionalization, and increased mortality. These non-cognitive signs and symptoms could be developed as inexpensive and readily accessible markers of AD progression in a clinical setting. Moreover, elucidating the underlying molecular mechanisms for these early clinical manifestations of AD may yield important insights into novel pathways affected in AD, which could lead to the development of important new therapeutic targets.

## Author Contributions

All authors participated in the study design, drafted/revised the manuscript, approved the final version and agreed to be accountable for all the aspects of the work.

## Conflict of Interest Statement

The authors declare that the research was conducted in the absence of any commercial or financial relationships that could be construed as a potential conflict of interest.
